# Dealing with missing delirium assessments in prospective clinical studies of the critically ill: a simulation study and reanalysis of two delirium studies

**DOI:** 10.1186/s12874-021-01274-1

**Published:** 2021-05-06

**Authors:** Rameela Raman, Wencong Chen, Michael O. Harhay, Jennifer L. Thompson, E. Wesley Ely, Pratik P. Pandharipande, Mayur B. Patel

**Affiliations:** 1 Vanderbilt University School of Medicine, 2525 West End Ave Suite 11, Nashville, TN USA; 2 Critical Illness, Brain Dysfunction, and Survivorship (CIBS) Center, Vanderbilt University Medical Center, Nashville, TN USA; 3 Department of Biostatistics, Epidemiology, and Informatics, Perelman School of Medicine, University of Pennsylvania, Philadelphia, PA USA; 4 Clinical Trials Methods and Outcomes Lab, Palliative and Advanced Illness Research (PAIR) Center, Perelman School of Medicine, University of Pennsylvania, Philadelphia, PA USA; 5 Division of Pulmonary and Critical Care, Department of Medicine, Perelman School of Medicine, University of Pennsylvania, Philadelphia, PA USA; 6 Tennessee Valley Veteran’s Affairs Geriatric Research Education Clinical Center (GRECC), Nashville, TN USA; 7 Division of Anesthesiology Critical Care Medicine, Department of Anesthesiology, Vanderbilt University Medical Center, Nashville, TN USA; 8 Section of Surgical Sciences, Departments of Surgery, Neurosurgery, & Hearing & Speech Sciences, Vanderbilt University Medical Center, Nashville, TN USA

**Keywords:** Summary exposure, Missing data, Critical care, Passive imputation, Active imputation, Longitudinal data, Long-term outcome, Delirium

## Abstract

**Background:**

In longitudinal critical care studies, researchers may be interested in summarizing an exposure over time and evaluating its association with a long-term outcome. For example, the number of days a patient has delirium (i.e., brain dysfunction) during their critical care stay is associated with the presence and severity of long-term cognitive problems. In large pragmatic trials and multicenter observational studies, particularly when electronic medical record data is used, the information on daily exposure status may be available at some time points and not at others. Model-based multiple imputation is a well-established, widely adopted method to deal with missing data. But the uncertainty around multiple imputation for summary exposure variables is whether the imputation is to be performed at the summary level or at the daily assessment level.

**Methods:**

We compare the following approaches to imputing and summarizing partially missing longitudinal data: 1) active imputation, where we impute the summary; 2) passive imputation, where we impute the daily missing data, and then compute the summary; 3) ad hoc methods where we assume all missing time points have the a) most or the b) least extreme value; and 4) complete case analysis where only participants with complete data are analyzed. These methods were applied under different missingness mechanisms, varying proportions of missingness, and association of missingness with an auxiliary variable using simulations that closely mirrors real-life critical care data to be relevant to real-world clinical practice. The performance of the approaches were compared using bias of the estimated coefficients, standard error of the estimate and coverage. We also apply these imputation strategies to two datasets in critical care.

**Results:**

Simulations show that all methods performed comparably when the proportion of missingness was small, indicating that in such instances, the gain over using any imputation model is minimal. But as the proportion of missingness increases, the passive imputation approach provides efficient and less biased estimates under the missingness at random and missingness completely at random mechanism.

**Conclusions:**

For longitudinal data where a summary exposure is of interest, we recommend practitioners adopting the passive imputation strategy.

## Background

In clinical research, longitudinal time-varying exposure for a participant is often condensed into a summary exposure. This is particularly common in critical care research, where the total duration of an event like mechanical ventilation, delirium (i.e., brain dysfunction), or other organ failure states are compared to assess the comparative effectiveness of interventions or prognosticate long-term morbidity and mortality [1]. Summarizing a longitudinal variable using one value is done to describe the participants’ overall exposure and when the goal is to correlate the exposure to a long-term outcome. Doing this also enables use of univariate approaches such as a t-test or a Wilcoxon rank-sum test to compare summaries between study groups [2].

Missing data is inevitable in clinical research. In a randomized clinical trial, participants may drop out due to adverse effects, lack of efficacy, or measurements may be missing due to death. In observational studies, especially ones that derive the data from the electronic medical record, missing data can be frequent. Summary variables are hard to compute when the daily assessments that form it are missing at specific time points. This poses the question of how to use all available data while reducing the chance that missing data will bias results.

The commonly adopted method for missing data is multiple imputation [3]. It involves assumptions regarding the missingness mechanism and the specification of an imputation model. Although the advantages of multiple imputation over complete case analyses are widely recognized, literature is limited on the performance of imputation strategies in the context of summary statistics computed from longitudinal data. The goal of this paper is to evaluate strategies to impute summary statistics, such as the duration of an exposure when daily assessments needed to compute it has missingness.

## Methods

### Study design

We compare several approaches: 1) active imputation, where we impute the summary; 2) passive imputation, where we impute the daily missing data, and then compute the summary; 3) ad hoc methods where we assume all missing time points have the a) most or the b) least extreme value; and 4) complete case analysis. Simulation studies are conducted closely mimicking the data structure from the Bringing to Light the Risk Factors and Incidence of Neuropsychological Dysfunction in ICU Survivors (BRAIN-ICU) study to compare the relative performance of these approaches to imputing and summarizing partially missing longitudinal data. In addition, we also illustrate the impact of each imputation technique using the BRAIN-ICU and the Traumatic Brain Injury (TBI) studies described below.

### Study population

Our study was motivated by two studies, a prospective NIH-funded observational study and a retrospective observational study with data extracted from electronic medical records.

#### Study 1

Bringing to Light the Risk Factors and Incidence of Neuropsychological Dysfunction in ICU Survivors (BRAIN-ICU) was an NIH-funded multi-center prospective cohort study, which enrolled 821 adults with respiratory failure or shock between March 2007 and May 2010. The primary goal of this study was to evaluate the association between the summary variable delirium duration over their critical care stay (defined as the number of days with delirium) and cognitive impairment (measured using the Repeatable Battery for the Assessment of Neuropsychological Status instrument-RBANS) at 3- and 12-months post study enrollment. Trained research personnel evaluated participants’ delirium status (a yes/no variable) daily until death, hospital discharge, or study day 30. Details on how delirium was assessed can be found in the primary study publication [4].

#### Study 2

The traumatic brain injury (TBI) study is a retrospective observational cohort of 4821 participants aged 16 years or older admitted between August 2006 and July 2012 with TBI requiring hospitalization with at least 1 day in the ICU. TBI was classified using the electronic medical record data based on ICD-9 diagnostic codes and included concussion without intracranial hemorrhage. One of this study’s goals was to evaluate the association between the summary variable delirium duration and Functional Independence Measure at discharge, a score that describes disability and burden of care among TBI survivors.

These two data sources are similar in that they both evaluate the association of a summarized exposure variable, delirium duration, and a long-term outcome. But they vary in the rigor by which the data was collected. As an NIH-funded observational cohort study, the outcomes and measures in BRAIN-ICU were prospectively collected in a consistent, systematic manner in accordance with the study protocol. On the contrary, the TBI study leveraged existing electronic health records where investigators typically have no control over the original collection of the data that was not captured for research purposes.

### Ethics approval

The BRAIN-ICU study was conducted at Vanderbilt University Medical Center and Saint Thomas Hospital in Nashville. The study protocol was approved by each local institutional review board and informed consent was obtained from all subjects for the original study. The TBI study involved retrospective data collected from the electronic health record system at Vanderbilt University Medical Center in Nashville and the study was approved by the Vanderbilt institutional review board. The data used in this manuscript from these two studies for secondary data analyses were deidentified. All methods were carried out in accordance with relevant guidelines and regulations. There were no experimental protocols in this study.

### Statistical methods

The computation of a summary exposure, such as the duration of an event, requires that the event be assessed and captured for each participant every day that they are alive and in the hospital. Multiple imputation is a well-established, widely adopted method to deal with missing data. The uncertainty around multiple imputation for summary exposure variables is whether the imputation is to be performed at the summary level or at the daily assessment level. Two approaches used in this scenario are active and passive imputation. Passive imputation, also known as ‘impute then transform,’ is where imputation is conducted at the assessment level, and then the summary exposure is computed [5]. The missing assessments are imputed B times using multiple imputation, and the summary statistic is computed for each of the B imputed datasets. The statistical model is then fit on the B imputed datasets, and a pooled estimate of the association effect is obtained using Rubin’s rules [3]. Concerns about passive imputation include misspecification of the imputation model that results in biased parameter estimates. An advantage is that this approach preserves and uses the observed assessments already collected for a participant. It also ensures that imputed values adhere to consistent relationships between variables and are therefore plausible, and it maintains the consistency among different transformations of the same data [6].

On the contrary, active imputation, also known as ‘just another variable’ approach, is where imputation is performed at the summary level [7]. Here, the summary is imputed using model-based imputation B times, the statistical model is fit on the B imputed datasets and estimates of effect are pooled. The drawback to this approach is that it ignores the non-missing assessments that already exist for a participant and do not take those into account during imputation of the summary. Active imputation also ignores the relationship that exists between the components and the summary statistic. More about passive and active imputation strategies for transformed variables such as interaction terms and quadratic terms can be read here [7–9].

Other strategies that have been used for missing data are complete-case and ad hoc methods that have little to no theoretical justification. Although it has been shown that they lead to biased and inefficient estimates when missingness is not at random, the ease of implementation and non-black-box nature of the ad hoc imputation process remains the primary reason for their frequent use in clinical research [10]. When individual assessments are missing, complete case analysis leads to summary exposures that remain undefined for some participants even though they may only have one time point missing. In such cases, a complete-case analysis would lead to the exclusion of participant-level data, thus reducing the sample size for analysis and translating to a biased estimate of the association between the exposure and the outcome.

Ad hoc approaches are generally varied in nature, such as carrying the last observation forward, substituting the group mean for missing data, and the approach adopted is often dependent on the discipline. Critical care studies’ common ad hoc strategy is to impute missing assessments with the worst or the best value and this has the advantage of preserving cases with missing data and thus maintaining the sample size. This approach is appealing due to its simplicity and accessibility, but it overestimates or underestimates the exposure and can potentially bias results. Another drawback is that potentially valuable information from auxiliary variables is not considered when imputing the missing assessment.

### Simulation study

#### Data generation process

The approaches discussed above were evaluated by randomly sampling from BRAIN-ICU using single-stage cluster sampling with the participant as the primary sampling unit. First, a simple random sample of participants is selected, and all the measurements (ICU days) from each of the selected participants are obtained. This ensures that the intra-cluster correlation typically observed in such data is maintained and closely mimics the data structure from BRAIN-ICU. Since missingness in the delirium status variable was minimal in this dataset (~ 3%), using a subset of 200 participants with complete data from this study as a foundation to simulate data enables us to closely mirror real-life critical care data and be relevant to real-world clinical practice. The data generation process is as follows:

1. Use single-stage cluster sampling to randomly sample 200 BRAIN-ICU participants with complete exposure data (daily delirium status) and an auxiliary (severity of illness represented using the Sequential Organ Failure Assessment (SOFA) scores) variable to create a participant-day dataset as shown in Table 1. Repeat to generate 1000 simulated datasets.
2. Generate the outcome, RBANS, for each participant as a function of delirium duration: *RBANS*_*i*_ = *α* + *β*_*del*_ ∗ *delirium duration* + *ϵ*_*i*_, where *ϵ*_*i*_~*N*(0, 12) and *β*_*del*_ = − 1.0.

**Table 1:** Illustrative data structure of simulated data

Participant ID	Study day	Delirium (Y/N)	Delirium duration	Daily SOFA score
1	1	Y	Undefined	12
1	2	Y		11
1	3	Missing		10
1	4	Y		10
1	5	N		10
2	1	Y	2	13
2	2	Y		13
3	1	N	0	14
4	1	Y	2	11
4	2	Y		11
4	3	N		11

#### Missing data process

Missing data was introduced in the 1000 simulated datasets by varying the proportion of missingness in the daily assessments, different missing mechanisms and the strength of association between the auxiliary variable and missingness as specified in Table 2. A total of 140 scenarios were studied.

**Table 2:** Missing data generation process for the simulation study by varying the proportion of missingness in the daily assessments, using different missing data mechanisms and varying the strength of association between the auxiliary variable and missingness

1. Proportion of missingness, *p*: 1%, 5%, 20%, 35% of participant-days missing exposure value 2. Types of missingness: a. Missing Completely at Random (MCAR): *p*% of the participant-days/assessments in each of the 1000 simulated datasets were removed completely at random. b. Missing at Random (MAR): MAR mechanism was simulated under a logistic regression model as a function of an auxiliary variable, the SOFA score, with varying correlations with missingness: $logit\ \left({missing}_{ij}\right)=\alpha +{\beta}_{SOFA_{ij}}\ast {SOFA}_{ij}$, where ${\beta}_{SOFA_{ij}}$ = 0.01, 0.1 and 0.2 representing weak, moderate and strong relationships with missingness. Here *α* was manipulated for different combinations of ${\beta}_{SOFA_{ij}}$ to generate the required proportion of missingness *p*. c. Missing Not at Random (MNAR): MNAR mechanism was generated under a logistic regression model with the probability of missingness having a weak, moderate, and strong association with the daily delirium status. *logit*(*missing*_*ij*_) = *α* + *β*_*del* _ *miss*_ ∗ *delirium*_*ij*_, where *β*_*del* _ *miss*_ took on values 0.1, 0.5 and 1.0 representing weak, moderate and strong relationships with missingness. Here *α* was manipulated for different combinations of *β*_*del* _ *miss*_ to generate the required proportion of missingness *p*.	

#### Analysis model

The association between the cognitive outcome (a normalized score) and delirium duration in each simulated dataset is analyzed using a linear regression model, *RBANS*_*i*_ = *α* + *β*_*del*_ ∗ *delirium duration* + *ϵ*_*i*_. This model does not adjust for any confounders and was identical across all strategies to focus solely on the performance of each of the approaches. For passive imputation, the auxiliary variable, daily SOFA score was used, while the mean SOFA score was used for active imputation. Multiple imputation was performed using the mice package in R and estimates are pooled using Rubin’s rules [3].

The metrics used to evaluate these approaches are:

1. Bias = Mean of the estimated coefficients from the 1000 replications - true coefficient = $E\left({\hat{\beta}}_{del}\right)-{\beta}_{del}$, where ${\hat{\beta}}_{del}$ represents the association between delirium duration and the outcome.
2. Mean of the standard error of the regression estimate = $E\left(\hat{\sigma}\right)$. It is optimal to have small standard errors, which translate to more precise estimates.
3. Coverage = Percent of 1000 simulations where the confidence interval of the estimate includes the true coefficient, *β*_*del*_. A coverage probability of 0.95 is considered optimal and indicates the percentage of confidence intervals that contain the true value of the estimate and the nominal confidence level.

## Simulation results

The performance of the approaches based on each metric by proportion of missing assessments are presented below. The results are stratified by the type of missingness and strength (weak, moderate, and strong) of the relationship of auxiliary variables with missingness.

### Bias

**Fig. 1:**
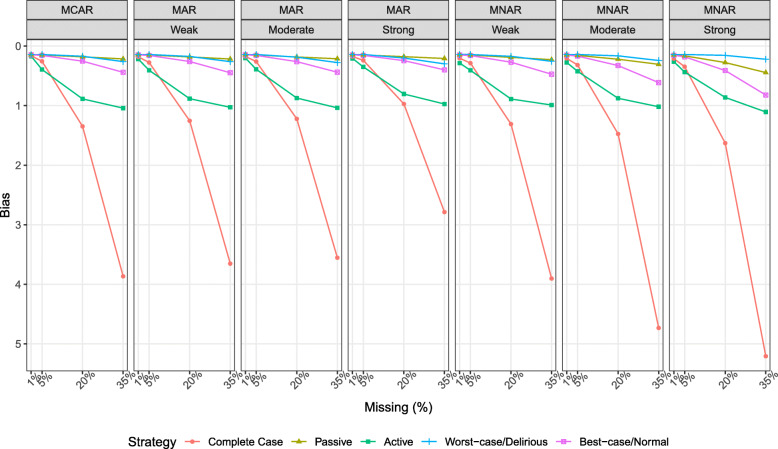
Bias in the association between delirium duration and the cognitive outcome for each imputation strategy stratified by the proportion of missingness, missingness mechanism, and association of missingness with the auxiliary variable. Results are derived from the analysis of 1000 simulated datasets

Figure 1 illustrates the bias in the association between delirium duration and the cognitive outcome for each imputation strategy. Bias increased with increase in the proportion of missingness for all imputation strategies. For low proportion of missingness (1, 5%), bias was maximum under MNAR for complete case and active imputation respectively. Passive imputation had the lowest bias compared to other strategies except when the strength of the relationship of the auxiliary variable with missingness under MNAR was strong. Unlike under MAR, bias increased for all strategies under MNAR as the relationship of the auxiliary variable with missingness went from weak to strong.

### Standard error estimate

**Fig. 2:**
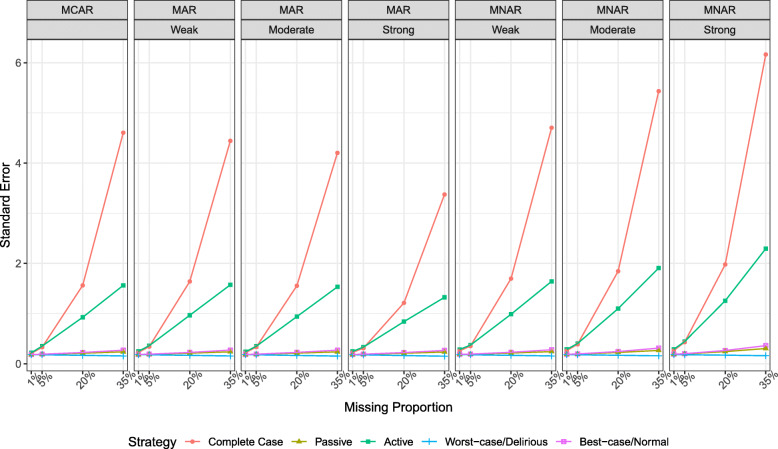
Standard error of the estimate between delirium duration and the cognitive outcome for each imputation strategy stratified by the proportion of missingness, missingness mechanism, and association of missingness with the auxiliary variable. Results are derived from the analysis of 1000 simulated datasets

Figure 2 shows that when missingness was minimal at 1%, regardless of the missingness mechanism, standard error estimates were similar and small for the passive and ad hoc strategies but slightly larger for active imputation and complete case. As the proportion of missingness increased, complete case and active imputation had the largest standard errors with the lowest standard error observed for passive imputation and the ad hoc strategy where the worst-case scenario was imputed. The standard errors for all methods increased with the strength of the relationship of the auxiliary variable under MNAR.

### Coverage

**Fig. 3:**
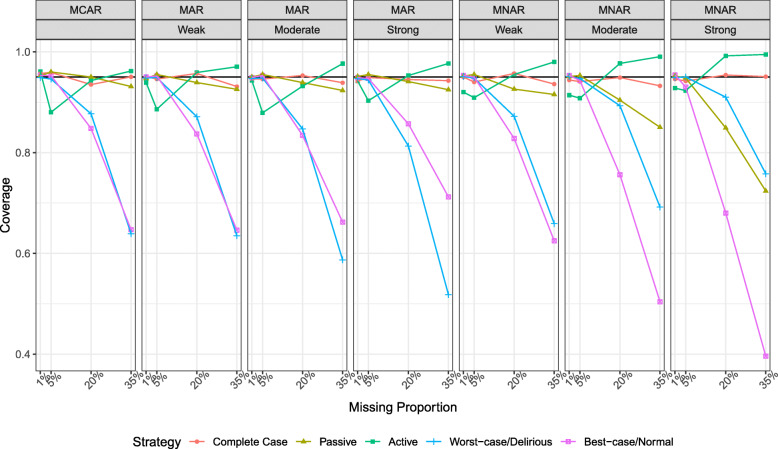
Coverage probability of the 95% confidence interval of the estimate between delirium duration and the cognitive outcome for each imputation strategy stratified by the proportion of missingness, missingness mechanism and association of missingness with the auxiliary variable. Results are derived from the analysis of 1000 simulated datasets. The solid black line represents 0.95

Figure 3 shows that for low proportion of missingness at 1%, coverage was 95% for passive and ad hoc strategies but lower for active and complete case. Coverage was consistently above 90% for passive imputation except when the strength of the relationship of the auxiliary variable with missingness under MNAR was moderate or strong. As the proportion of missingness increased, coverage was always acceptable, above 93%, for the complete case regardless of the missingness mechanism, but consistently inadequate for the ad hoc strategies, potentially leading to excessive type I errors. For example, at 35% of assessments missing, and MNAR, the best-case ad hoc strategy provided only 40% coverage.

### Application to motivating datasets

We illustrate these strategies using the BRAIN-ICU and TBI datasets with 3 and 32% of the daily assessments missing, respectively. At a participant-level, this translated to 28 and 62% of participants having partially missing assessments. We fit a linear regression model with the duration of delirium on the continuous outcome variables - cognitive impairment as measured using the Repeatable Battery for the Assessment of Neuropsychological Status instrument and disability and burden of care using the Functional Independence Measure at discharge, respectively. Though in reality we adjust for confounders in the model, here we fit the regression model with only delirium as the exposure for illustrative purposes and to focus on the comparison of strategies. The imputation models included a potential auxiliary variable, the daily SOFA score, to include information on missingness of the assessments.

**Fig. 4:**
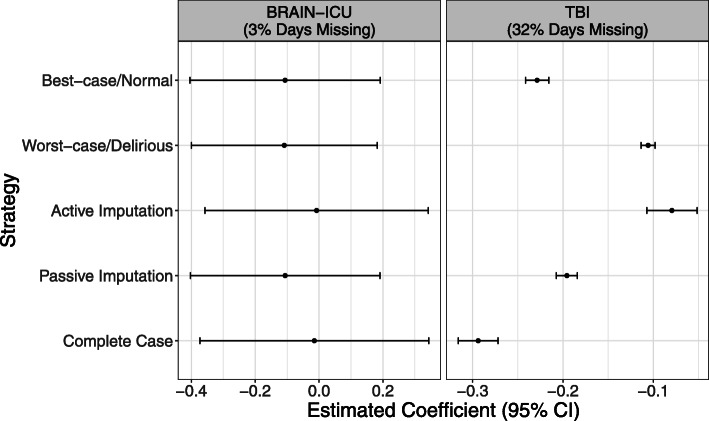
Estimated coefficients and the corresponding 95% confidence intervals using different imputation strategies, illustrated on the BRAIN-ICU and TBI studies

Figure 4 presents a comparison of the estimated association coefficient and 95% confidence intervals for the BRAIN-ICU and the TBI study using different strategies. For the BRAIN-ICU data, where only 3% of the daily assessments were missing, the estimated exposure coefficient and corresponding 95% CIs were similar between the passive imputation and ad hoc approaches. The estimated regression coefficient and 95% CI’s for complete case and active imputation were also similar but closer to the null with larger standard errors than the other approaches. For the TBI study, with 32% of the daily assessments missing, the estimated exposure coefficient range was wide (− 0.29 to − 0.08). As in the BRAIN-ICU data, standard errors were highest for the complete case and active imputation. The difference in these strategies’ performance between the two datasets emphasizes how much the proportion of missingness influence the estimates obtained using different methods.

## Discussion

In this study, we investigated five strategies for imputing missing longitudinal components of a summary exposure variable. Our simulation study showed that for a low proportion of missingness, the bias in the coefficients when using the passive and ad hoc strategies was minimal, irrespective of the missingness mechanism. Passive imputation also produced efficient estimates and had reasonable coverage as compared to other methods. As expected, and shown in the literature, complete case analysis was associated with severely biased estimates as the proportion of missingness increased [11, 12]. The ad hoc strategies provided estimates that were less biased and more efficient than the active and complete case analysis. Still, coverage was consistently low even under MCAR, potentially leading to excessive Type I errors.

Several papers have compared passive and active imputation strategies when computing exposure variables with missing components. Wagstaff [13] and Morris [14] looked at strategies to use BMI as an exposure when one of the components, weight, or height was missing. They found that under MCAR, both active and passive imputation performed similarly and that under MAR, active imputation was favored slightly. Others found that passive imputation performed better than active imputation when imputing an incomplete composite categorical variable [15, 16]. Similar examples have included non-linear effects and interaction terms where it has been shown that active imputation leads to less biased estimates than passive imputation, especially when missingness is completely at random [5, 8]. Our simulations showed that active imputation estimates were severely biased, inefficient and had low coverage. The difference between previous findings and our simulations lie in the structure of our dataset. Previous work considered cases where the summary exposure is a function of variables collected at the same time point versus ours is more complex and compute a summary exposure collected over time. For longitudinal datasets, active imputation is performed at the participant level and instead of the daily auxiliary variable, mean of the auxiliary variable over time is used in the imputation models. This probably is the reason for biased and less efficient estimates under active imputation. On the contrary, under MCAR and MAR, passive imputation was associated with the lowest bias and lower standard errors regardless of the missingness proportion. When using passive imputation techniques, the inclusion of daily auxiliary variable in the imputation model probably helps in increasing the precision and reducing the bias of parameter estimates as noted in previous studies [17–19].

Our simulation studies also revealed that as the proportion of missing daily assessments increased (to 50%, a scenario which is not presented in this paper for brevity), a complete strategy was often impractical and unviable. For example, imposing 20% missing participant-days in a sample size of 200 participants observed for 5 days each translates to 200 missing assessments. These 200 missing observations could possibly all be from different participants leading to missing exposures for every single participant in the sample. Under this scenario, active imputation would also involve imputing the summary for every participant, which might explain the high standard errors and biased estimates. Scenarios like this occurred in our simulated datasets, indicating that active imputation strategy may be an unsuitable method when missing assessments are extremely high. Although our simulations did not consider the effect of increasing the study sample size, it can be inferred from our results that in such studies, efficiency of our estimates depend on not just the total number of participants in the study, but the proportion of missing patient-days with respect to the total number of patients. Hence studies with larger sample sizes but lower proportion of missing patient-days would possibly have better properties as long as the missingness didn’t increase proportionally with sample size.

Our simulations could be expanded to include other methods to impute summary variables, like those designed for multilevel imputation incorporating the intra-cluster correlation. But since it is recommended that the imputation model be consistent with the analytic model [20, 21] and our analytic model was single-level versus a mixed-effects model, we didn’t consider generalized linear mixed-effects imputation methods. Van Buuren differentiates passive imputation from the impute-then-transform method where for the former, the computation of the summary variable is done on-the-fly within the imputation algorithm, which potentially removes the bias due to not including the dependencies between the variables [6]. When transforming variables using components collected at the same time point, the relationship between the computed variable and its components can be incorporated in the mice package in R. But this is not easily transferred and not straightforward to implement when working with unbalanced longitudinal data. Hence in this paper, passive imputation is the same as the impute-then-transform method. Our simulation parameters were generated based on a dataset in critical care research. Hence our findings may not always be generalizable or translate seamlessly to all longitudinal data where summary exposures are missing.

In summary, all methods performed comparably when the proportion of missingness was extremely small at 1% indicating that when the proportion of missingness is so low, the gain over using all imputation models are minimal. But as the proportion of missingness increased, passive imputation strategy performed better with respect to bias, efficiency and coverage compared to the other methods. Even under MCAR, as the proportion of missingness increased, active imputation and complete case yielded estimates that were severely biased and inefficient. But the use of passive imputation over ad hoc strategies did not provide any advantage with regards to bias, efficiency or coverage. In our NIH-funded observational study, it is likely the case that the missing data are MCAR, but MNAR in data extracted from the electronic medical record. Our work re-emphasizes that although the electronic health records present a wealth of data, it is important to address missingness to make valid inferences. This work and findings can also be extended to composite endpoints in clinical trials when some components may be missing [16]. Missing data is a threat to the validity of results and the appropriate strategy depends on several factors such as the proportion of missingness, structure of the data, availability of informative covariates. It is hard to provide concrete guidelines, and we encourage sensitivity analyses to evaluate the robustness of findings.

## Conclusion

In summary, our simulations show that all methods performed comparably when the proportion of missingness was small, indicating that in such instances, the gain over using any imputation model is minimal. But as the proportion of missingness increases, the passive imputation approach provides efficient and less biased estimates under the missingness at random and missingness completely at random mechanism. For longitudinal data where a summary exposure is of interest, we recommend practitioners adopting the passive imputation strategy. Since the appropriate strategy depends on several factors, we also encourage sensitivity analysis to evaluate the robustness of findings.

## Data Availability

Software code for the simulation studies are available from the corresponding author upon request.

## Abbreviations

BRAIN-ICU: Bringing to Light the Risk Factors and Incidence of Neuropsychological Dysfunction in ICU Survivors
RBANS: Repeatable Battery for the Assessment of Neuropsychological Status
TBI: Traumatic Brain Injury
ICU: Intensive Care Unit
SOFA: Sequential Organ Failure Assessment
MCAR: Missing Completely at Random
MAR: Missing at Random
MNAR: Missing Not at Random
